# Clinical profile and outcomes of dematiaceous fungal keratitis - data from a secondary center in rural North India

**DOI:** 10.1186/s12348-025-00555-w

**Published:** 2025-11-26

**Authors:** Sonali Mehta, Nikunj Vinodbhai Patel, Arpan Gandhi, Isha Chaudhari, Atanu Majumdar, Umang Mathur, Manisha Acharya

**Affiliations:** 1 Department of Cornea, Dr. Shroff’s Charity Eye Hospital, 5027, Kedarnath Lane, Daryaganj, New Delhi, 110002 India; 2 Department of Laboratory Services, Dr. Shroff’s Charity Eye Hospital, New Delhi, India; 3 Department of Public Health, Dr. Shroff’s Charity Eye Hospital, New Delhi, India

## Abstract

**Purpose:**

To evaluate the clinical course, factors contributing to healing, and outcomes of medically treated dematiaceous fungal keratitis (DFK) at a rural secondary eye care center in North India.

**Methods:**

This retrospective, electronic medical record-based study was conducted over one year at a secondary care center. Patients of all age groups with culture-proven DFK who completed follow-up until corneal healing were included. Demographic, clinical, and microbiological data were analyzed. All patients received therapeutic debridement and natamycin monotherapy. Voriconazole was added in non-responders, and oral antifungals were used for corneal melt or posterior segment involvement. Cases requiring therapeutic keratoplasty (TPK) or evisceration were classified as poor outcomes.

**Results:**

Hundred and eight eyes were included in the study. The mean age was 43.2 years, with 78.7% males. Trauma, most commonly from sugarcane leaves, was reported in 80.6%. Ulcers were mild in 50%, moderate in 39.8%, and severe in 10.2%. Half were small in size; 89.8% were superficial, while 5.6% showed full-thickness involvement. Neurotrophic-like defects were seen in 48.5% and significantly associated with ulcer size (*p* = 0.004). Healing occurred within 2 weeks in 44.5% and within 4 weeks in 33.7%. Delayed healing (21.8%) correlated with larger, deeper ulcers, hypopyon, and neurotrophic-like defects. TPK/evisceration (12%) was significantly associated with prior steroid use, vascularization, hypopyon, and corneal melt (*p* < 0.05).

**Conclusion:**

Despite prolonged healing, DFK has a high resolution rate with appropriate medical therapy, minor procedures, and complete debridement. Poor prognostic factors include large and deep infiltrates, hypopyon, and prior steroid use.

## Introduction

Infectious keratitis (IK) is the leading cause of corneal blindness in developing countries. In India, Fungal keratitis (FK) shares a 15–25% prevalence of IK [1]. Fungi are usually present in soil; trauma with vegetative matter is the most common etiological factor for FK. Most of the Indian population (69%, India Census 2011) resides in rural areas [2]. Nearly 55% of the population is engaged in agriculture [3]. This population is highly susceptible to fungal infections [4].

Nearly 40% of FK patients are known to have poor outcomes [5]. However, recent reports from South India suggest a higher healing rate (80%) for keratitis associated with dematiaceous fungi [6, 7].

The different pathophysiological patterns of the major sub-groups of fungi form the basis of the difference in the healing rates. Long-standing *Aspergillus* and *Fusarium* species (spp). infect an already compromised ocular surface with a higher tendency for deeper penetration. In contrast, dematiaceous fungi (DF) colonize the ocular surface with a plane of separation from the underlying stroma in the form of an infectious plaque [8]. This leads to a slow and indolent course without much deeper penetration into the stroma. Histologically, this plaque consists of aggregated fungal filaments with the destruction of the underlying stroma [8]. Therefore, theoretically, complete plaque removal hastens the recovery.

The literature contains a limited number of studies profiling the clinical course and outcomes. In this study, we aimed to analyze the clinical course, factors affecting healing, and eventual outcomes of medically treated cases of dematiaceous fungi keratitis (DFK) from a secondary care center in North India. This study is also unique as it is based in a rural setting, serving a predominantly agrarian population prone to vegetative trauma and infectious keratitis. In our hospital these patients have access to cornea care directly at the secondary center and even at the vision centers (primary center). This distinguishes our model from those based in urban tertiary centers. By providing both specialty cornea services with microbiology support close to the point of need, we enable earlier diagnosis and intervention [9]. This is especially important for less virulent pathogens such as DF, which may respond to treatment without requiring referral to a higher center.

## Materials and methods

This retrospective electronic medical record system-based study was conducted at a secondary care center in North India over one year (January 2023 - December 2023). Institutional review board of Dr. Shroff’s Charity Eye Hospital approved the study (IRB/2025/SEP/16), and the study adhered to the tenets of the Declaration of Helsinki.

### Inclusion and exclusion criteria

The study included patients of all age groups with culture-proven DFK. We excluded patients who were lost to follow-up before complete healing.

Demographic details of each patient, history including inciting trauma, onset, duration, and progression of symptoms, and previous ocular treatment history were noted. Clinical features, including the size of the plaque and the infiltrate, the presence of pigmentation, hypopyon, thinning, and vascularization, were noted. Slit lamp imaging was done on every visit. Associated posterior segment infection was evaluated with a dilated fundus evaluation or an ultrasound B scan when the fundus could not be visualized. The severity of the ulcer was determined based on a combination of the size and depth of the infiltrates as follows: mild (< 2 mm size and/or < 20% depth), moderate (2–5 mm size and/or 20% − 50% depth), or severe (>5 mm size and/or >50% depth) [10]. All ulcers were also independently graded based on size and depth.

### Management

On the first visit, a corneal scraping on slit lamp under topical anesthesia was performed to obtain a sample for microbiological testing. The sample was smeared on two slides and plated on blood agar, chocolate agar, and Sabouraud dextrose agar (SDA). The slides were utilized for Gram stain and 10% potassium hydroxide wet mount. Dematiaceous fungi were identified by.

their colony characteristics on solid media and by the morphological appearance of the spores in lactophenol cotton blue stain or KOH mount. An isolate was considered dematiaceous if fungal colonies revealed dark brown, greyish black or olive-black pigmentation, and lactophenol cotton blue mount or KOH mount from the culture revealed black or brown pigmented septate hyphae, conidia, or both.

Once the smears revealed fungal hyphae, as per institutional protocol, every patient underwent either a therapeutic debridement or a plaque removal on the slit lamp under topical anesthesia (0.5% proparacaine eye drops). For plaque removal, after the instillation of topical anesthesia, the plaque was held with toothed forceps, and a superficial keratectomy was done with a number 15 blade at the plane of separation from the underlying stroma. Initially, every patient received monotherapy with topical natamycin eye drops 5% hourly and a cycloplegic eye drop thrice a day. Patients were reviewed every 72 h. At each follow-up, debridement or plaque removal was repeated till the signs of healing were noted.

The presence of a “neurotrophic-like epithelial defect” after debridement or plaque removal was noted on subsequent follow-up.

Healing was defined as a reduction in the size/density of the infiltrate, epithelial defect, and hypopyon. This was determined by comparing electronic medical records and anterior segment photos from slit lamp imaging with previous visit details. Worsening was defined as an increase in the size of the infiltrate, formation, or an increase in the size of hypopyon, increased thinning, or presence of a corneal melt. In case of worsening or a status quo condition, topical voriconazole 1% hourly was added to the monotherapy regimen after 1 or 2 weeks. Oral antifungal medications were indicated in cases with corneal melt, posterior segment spread, or worsening despite two topical antifungal medications. Minor procedures performed were either intracameral (ICV) or intrastromal (ISV) injections of voriconazole 50 µg/milliliter, tarsorrhaphy, and tissue adhesive with bandage contact lens (TA + BCL). Therapeutic Penetrating Keratoplasty (TPK) was advised when there was worsening or associated corneal perforation or melt not amenable to TA + BCL, corneal limbal involvement, or in cases with associated endophthalmitis.

Complete healing was defined as the absence of conjunctival congestion, epithelial defect, corneal infiltrate, and hypopyon. Cases that achieved complete healing with medical therapy alone or with minor procedures were classified as having good outcomes. Poor outcomes were defined as patients who required a major surgical intervention, including a TPK or an evisceration.

Eyes with good outcomes were further divided based on the time taken for complete healing (< 2 weeks, 2–4 weeks, > 4 weeks).

The primary outcome was correlating the time to heal with various inciting factors, clinical features, and treatment options. The secondary outcome was to correlate the size and depth of the ulcer separately with the outcomes of the ulcer.

### Statistical analysis

Comparisons were conducted using Fisher’s exact test to evaluate associations between treatment history, trauma history, clinical characteristics, and time-to-heal metrics. A significance threshold of 0.05 was applied. Odd’s ratio was calculated and descriptive statistics presented with their corresponding 95% confidence intervals. All statistical analyses were performed using R version 4.4.2.

## Results

During the study period, 445 cases of culture-proven fungal keratitis were identified. Of these, 48.5% of cases were caused by DF, 44% by *Fusarium* sp., and 7.4% by *Aspergillus* sp. Of the 216 cases diagnosed with DFK, data from 108 eyes that met the inclusion criteria were included for statistical analysis.

The mean age of the population was 43.16 years (range: 11–96 years). Eighty-five patients were male, while 23 patients were female.

The mean duration of symptoms was 11.41 days (range: 2–60 days). A history of trauma was present in 80.6% of patients (*n* = 87), most commonly caused by sugarcane leaves (44.4%, *n* = 48). Other sources included paddy (9.3%, *n* = 10), unspecified vegetative matter (17.6%, *n* = 19), dust (4.6%, *n* = 5) and miscellaneous objects (4.6%, *n* = 5).

### Previous treatment history

Overall, 38.9% of the eyes (*n* = 42) were treatment-naïve, while 61.1% (*n* = 66) had a history of previous treatment. Among the previously treated eyes, treatment was documented in 63.6%, while 36.4% had received treatment that was not documented. Of those with available treatment records, 97.6% had been treated with antibiotics, 42.9% had received antifungal treatment, and steroids were used in 33.3% of cases .

### Clinical features

Table 1 shows the clinical features of DFK observed in the study. The severity of the ulcers, combining size and depth, was graded as mild in 54 eyes (50%), moderate in 43 eyes (39.8%), and severe in 11 eyes (10.2%). The most common feature observed was infiltrate, present in 56 cases (51.9%), followed closely by plaque formation in 52 cases (48.1%). Other notable findings included pigmentation in 32 cases (29.6%), hypopyon in 20 cases (18.5%), corneal melt in 7 cases (6.5%), and vascularization in 6 cases (5.6%). Endophthalmitis was rare, which occurred in only one case (0.9%).

**Table 1:** Clinical features of dematiaceous keratitis

	Number of cases (*N* = 108)	% (95% CI)
Infiltrate	56	51.9% (42.43% − 61.28%)
Plaque	52	48.1% (38.72% − 57.57%)
Pigmentation	32	29.6% (21.02% − 38.24%)
Hypopyon	20	18.5% (11.19% − 25.84%)
Melt	7	6.5% (1.84% − 11.12%)
Vascularization	6	5.6% (1.24% − 9.88%)
Endophthalmitis	1	0.9% (0% − 2.73%)
**Severity Overall**		
Mild	54	50% (40.57% − 59.43%)
Moderate	43	39.8% (30.58% − 49.05%)
Severe	11	10.2% (4.48% − 15.89%)
**Ulcer Size**		
Small	54	50% (40.57% − 59.43%)
Medium	45	41.7% (32.37% − 50.96%)
Large	9	8.3% (3.12% − 13.55%)
**Ulcer Depth**		
Superficial	97	89.8% (84.11% − 95.52%)
Mid-stromal	3	2.8% (0% − 5.88%)
Deep-stromal	2	1.9% (0% − 4.39%)
Perforated	6	5.6% (1.24% − 9.88%)

**Fig. 1:**
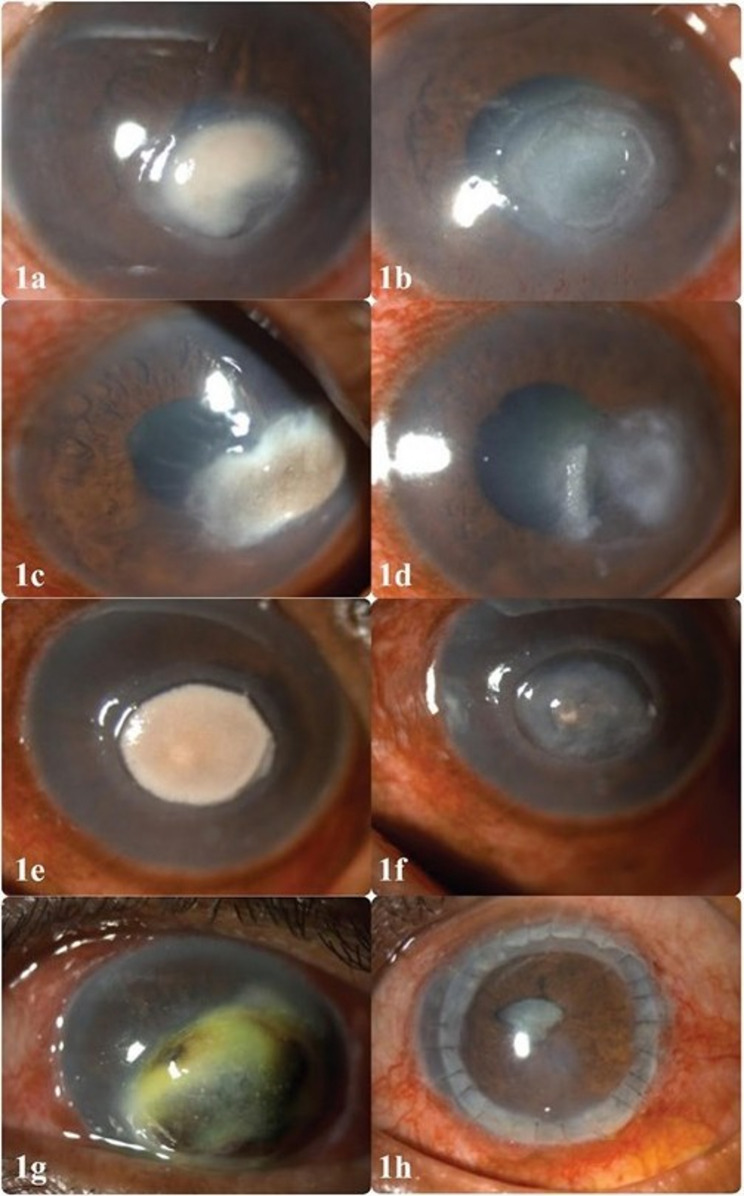
The image shows various presentations of dematiaceous fungal keratitis. **a** shows mild severity at presentation, **c** and **e** show moderate severity keratitis, and **g** shows severe keratitis with corneal melt. Images **b**, **d**, and **f** show corresponding images of the patients (**a**, **c** & **e**, respectively) after complete plaque removal. Image **h** shows the post-keratoplasty image of the severe keratitis case

Half of the cases (*n* = 54) were classified as mild, 43 cases (39.8%) were moderate, and 11 (10.2%) were severe. Based on the ulcer size, 54 cases (50%) (Fig. 1a) were small, 45 cases (41.7%) were medium (Fig. 1c and e), and 9 cases (8.3%) were large. Based on depth, superficial ulcers were the most prevalent, found in 97 cases (89.8%). Deeper stromal involvement was less common, with mid-stromal ulcers reported in 3 cases (2.8%) and deep-stromal ulcers in 2 cases (1.9%). Perforation occurred in 6 cases (5.6%) (Fig. 1g).

### Treatment and outcomes

Ninety-six cases (88.9%) were managed medically, five cases (4.6%) required minor procedures, and seven cases (6.5%) underwent TPK or evisceration (Table 2). Patients who had received steroids had a significantly higher rate of TPK or evisceration (21.4%, *p* = 0.032) compared to those previously treated without steroids (Fig. 1h).

**Table 2:** Medical and surgical management of DFK cases

	Medical management	Minor procedures involved	TPK/ Evisceration	Total	OR	*p*-value (Fisher’s Exact Test)
Total number of cases	96 (88.9)	5 (4.6)	7 (6.5)	108		
**History of trauma**						
No history of trauma	15 (71.4)	3 (14.3)	3 (14.3)	21	3.46	0.016
History of trauma	81 (93.1)	2 (2.3)	4 (4.6)	87	Ref	
**Treatment History**						
Treatment naïve	57 (86.4)	3 (4.5)	6 (9.1)	66	4.10	0.399
Previously treated	39 (92.9)	2 (4.8)	1 (2.4)	42	Ref	
**Previous treatment details (includes only those treated and treatment documents were available)**						
Antifungal	17 (94.4)	1 (5.6)	0 (0.0)	18	Ref	0.379
Antibiotic	36 (87.8)	2 (4.9)	3 (7.3)	41	-	1.000
Steroids	11 (78.6)	0 (0.0)	3 (21.4)	14	-	0.032
**Clinical Characteristics**						
Infection type: Infiltrate	47 (83.9)	2 (3.6)	7 (12.5)	56	Ref	0.019
Infection type: Plaque	49 (94.2)	3 (5.8)	0 (0.0)	52	0.00	
Melt	0 (0.0)	1 (14.3)	6 (85.7)	7	42.00	0.000
Endophthalmitis	0 (0.0)	0 (0.0)	1	1	-	0.111
Pigmentation	28 (87.5)	2 (6.2)	2 (6.2)	32	0.47	0.879
Hypopyon	13 (65.0)	2 (10.0)	5 (25.0)	20	2.33	0.001
Vascularization	2 (33.3)	0 (0.0)	4 (66.7)	6	14.00	0.000
**Severity Overall**						
Mild	54	0 (0.0)	0 (0.0)	54	Ref	0.000
Moderate	39 (90.7)	4 (9.3)	0 (0.0)	43		
Severe	3 (27.3)	1 (9.1)	7 (63.6)	11	-	
**Ulcer size**						
Small	54	0 (0.0)	0 (0.0)	54	Ref	0.000
Medium	39 (86.7)	5 (11.1)	1 (2.2)	45	-	
Large	3 (33.3)	0 (0.0)	6 (66.7)	9	-	
**Ulcer depth**						
Superficial	93 (95.9)	4 (4.1)	0 (0.0)	97	Ref	0.000
Mid stromal	3	0 (0.0)	0 (0.0)	3		
Deep stromal	0 (0.0)	1 (50.0)	1 (50.0)	2	-	
Perforated	0 (0.0)	0 (0.0)	6	6	-	

* TPK/ evisceration has been considered a poor outcome. The hyphen (“-“) represents those conditions where all cases had a poor outcome or all reference cases had a good outcome, and hence, ORs were mathematically undefined. However, in reality, this implies a very high odds ratio

None of the cases presenting with a plaque required a surgical intervention as compared to 12.5% of cases presenting with an infiltrate (*p* = 0.019). Nearly all cases with corneal melt required a surgical procedure, with an 85.7% rate of TPK or evisceration (*p* < 0.001). The presence of hypopyon (25.0%, *p* = 0.001) and vascularization (66.7%, *p* < 0.000) also significantly increased the likelihood of TPK or evisceration.

All cases classified as mild or moderate severity were successfully managed medically or with minor procedures. Severe cases, however, had a TPK or evisceration rate of 63.6% (*p* ≤ 0.001).

Larger ulcer sizes and deeper stromal penetration had TPK or evisceration rates of 66.7% (*p* ≤ 0.001) and 95.9% (*p* ≤ 0.000), respectively, compared to their smaller and less invasive counterparts.

Of the medically treated 101 eyes, 49 (48.0%) exhibited neurotrophic-like defects (Table 3).

**Table 3:** Difference in clinical features between cases with and without neurotrophic type defects

	No neurotrophic type defects	Presence of neurotrophic type defects	*p*-value (Fisher’s exact test)
Entire sample	52 (51.5%)	49 (48.5%)	
95% CI	(42.26% − 61.66%)	(38.34% − 57.74%)	
**Overall severity**			
Mild	34 (63.0)	20 (37.0)	0.069
Moderate	17 (39.5)	26 (60.5)	
Severe	2 (40.0)	3 (60.0)	
**Ulcer Size**			
Small	35 (64.8)	19 (35.2)	0.004
Medium	17 (38.6)	27 (61.4)	
Large	0 (00.0)	3 (100.0)	
**Ulcer depth**			
Superficial	50 (51.5)	47 (48.5)	0.801
Mid stromal	1 (33.3)	2 (66.7)	
Deep stromal	1 (100.0)	0 (0.0)	

Figure 1b and d, and f show the neurotrophic-like defect after complete therapeutic debridement. The presence of neurotrophic-like defects was significantly associated with ulcer size (*p* = 0.004), being observed in 100% (3 cases), 61.4% (27 cases), and 35.2% (19 cases) of large, medium, and small ulcers, respectively. No significant association was found between neurotrophic defects and ulcer depth or the overall severity of keratitis.

### Time to heal

Of the 101 medically treated cases, 44.5% resolved within 2 weeks, 33.7% resolved within 3–4 weeks, and 21.8% required more than 4 weeks to resolve. As described in Table 4, a previous history of treatment did not influence the time to heal.

**Table 4:** Time to heal

	Within 2 weeks	3–4 weeks	Beyond 4 weeks	Total	OR* (poor outcome)	*p*-value (Fisher’s exact test)
Total number of cases	45 (44.5)	34 (33.7)	22 (21.8)	101 (100.0)		
No history of trauma	6 (33.3)	4 (22.2)	8 (44.4)	18	3.94	0.052
History of trauma	39 (47.0)	30 (36.1)	14 (16.9)	83	Ref	
Treatment History						
Received previous treatment	25 (41.7)	21 (35)	14 (23.3)	60	1.26	0.77
Treatment naïve	20 (48.8)	13 (31.7)	8 (19.5)	41	Ref	
Types of Drugs (prior treatment)						
Anti-fungal	6 (33.3)	9 (50.0)	3 (16.7)	18	Ref	0.297
Antibiotic	17 (44.7)	13 (34.2)	8 (21.1)	38	1.33	1.00
Steroids	6 (54.5)	3 (27.3)	2 (18.2)	11	1.11	0.913
Clinical Characteristics						
Infection type: Infiltrate	21 (42.9)	18 (36.7)	10 (20.4)	49	Ref	0.843
Infection type: Plaque	24 (46.2)	16 (30.8)	12 (23.1)	52	1.17	
Melt	0 (0.0)	0 (0.0)	1	1	-	0.218
Pigmentation	13 (43.3)	9 (30.0)	8 (26.7)	30	1.42	0.752
Hypopyon	3 (20.0)	5 (33.3)	7 (46.7)	15	3.41	0.028
Vascularization	2 (66.7)	0 (0.0)	1 (33.3)	3	1.95	0.696
Severity Overall						
Mild	31 (57.4)	17 (31.5)	6 (11.1)	54	Ref	0.000
Moderate	14 (32.6)	17 (39.5)	12 (27.9)	43	3.10	
Severe	0 (0.0)	0 (0.0)	4	4	-	
Ulcer size						
Small	32 (59.3)	16 (29.6)	6 (11.1)	54	Ref	0.000
Medium	13 (29.5)	18 (40.9)	13 (29.5)	44	3.35	
Large	0 (0.0)	0 (0.0)	3	3	-	
Ulcer depth						
Superficial	44 (45.4)	33 (34.0)	20 (20.6)	97	Ref	0.414
Mid stromal	1 (33.3)	1 (33.3)	1 (33.3)	3	1.93	
Deep stromal	0 (0.0)	0 (0.0)	1	1	-	
No neurotrophic ulcer	29 (55.8)	18 (34.6)	5 (9.6)	52	Ref	0.005
Neurotrophic ulcer formed	16 (32.7)	16 (32.7)	17 (34.7)	49	4.99	

* Healing time beyond four weeks has been considered a poor outcome. The hyphen (“-“) represents those conditions where all cases had a poor outcome or all reference cases had a good outcome, and hence, ORs were mathematically undefined. However, in reality, this implies a very high odds ratio

Hypopyon cases (*n* = 15) were more likely to resolve beyond 4 weeks (46.7%), with a significant p-value of 0.028. Other clinical characteristics, such as infiltrate or plaque, vascularization, and pigmentation, showed no significant differences in resolution time.

Mild cases (*n* = 54) resolved significantly faster, with 57.4% resolving within 2 weeks. Only 11.1% of mild cases required more than 4 weeks to resolve. Moderate cases (*n* = 43) showed a higher proportion resolving within 3–4 weeks (39.5%). All severe cases (*n* = 4) required more than 4 weeks to resolve, with a significant association (*p* < 0.001).

Smaller lesions (*n* = 54) resolved faster, with 59.3% resolving within 2 weeks, while medium lesions (*n* = 44) showed a higher proportion resolving within 3–4 weeks (40.9%). All large lesions (*n* = 3) required more than 4 weeks to resolve, with a significant p-value (< 0.001). Superficial lesions (*n* = 97) had the fastest resolution, with 45.4% resolving within 2 weeks whereas deep stromal lesions (*n* = 2) required more than 4 weeks to resolve. There was no significant association between stromal depth and resolution time (*p* = 0.414).

**Fig. 2:**
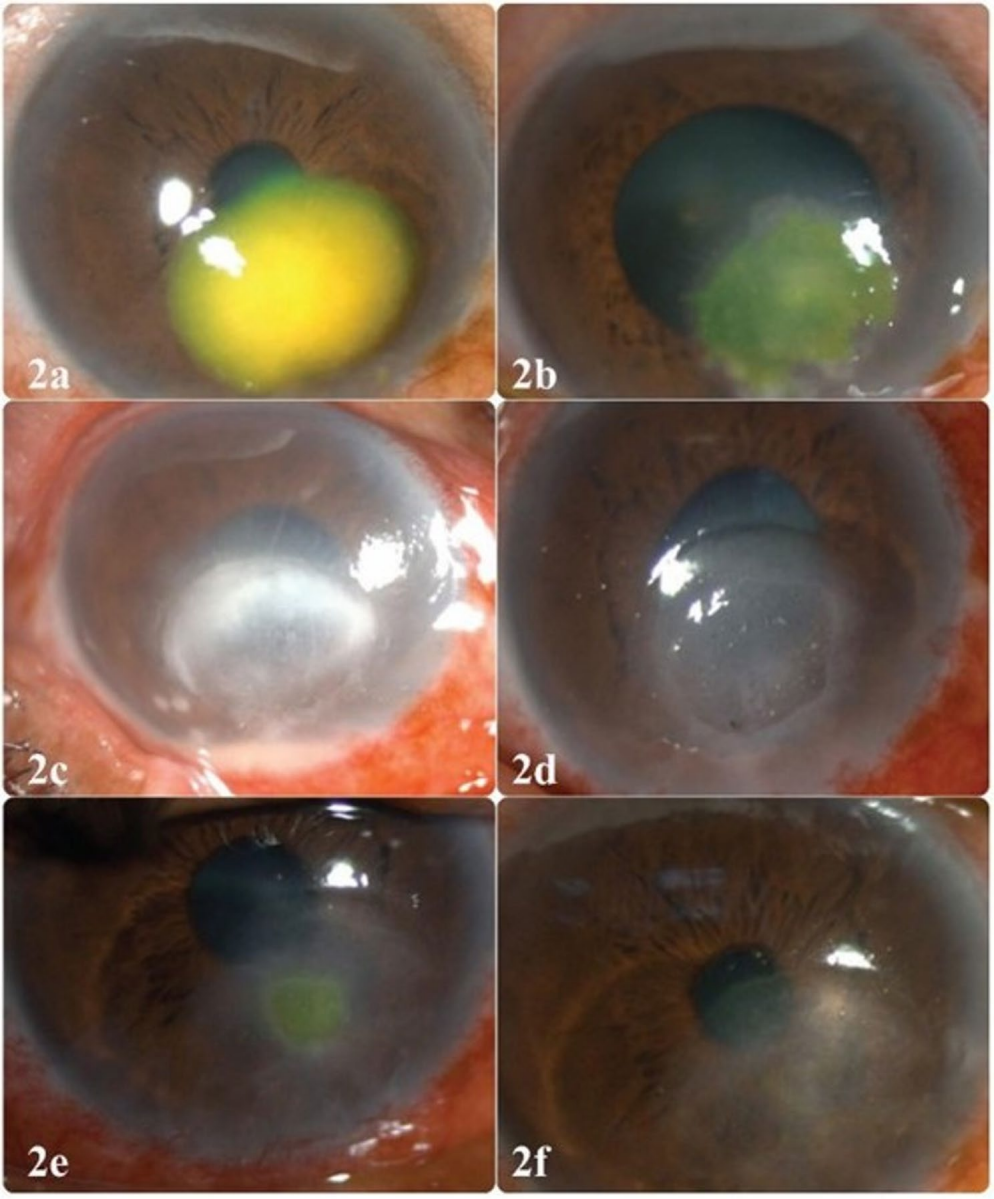
The series of images show the clinical course of medically treated moderate dematiaceous keratitis which got healed over a period of four weeks. Image 2**d** shows the typical ‘neurotrophic-like epithelial defect’ with minimal infiltrates

Formation of neurotrophic-like ulcers was associated with longer healing periods (Fig. 1b, d and f). Cases without this feature (*n* = 53) resolved faster (55.8% within 2 weeks) compared to those with neurotrophic ulcers, showing a significant association (*p* = 0.005). Figure 2 (a-f) shows an example of the clinical course and timeline of ulcer healing with formation of a neurotrophic-like epithelial defect.

## Discussion

DFK is responsible for a significant proportion of all FK, ranging from 15 to 25% [6, 7]. The major source of DF is soil and agriculture-associated trauma. Thus, the rural population is more susceptible to DFK. Being the primary point of contact, the data from a peripheral rural center is likely to reflect the true burden of the disease. This is reflected by the higher prevalence of DFK in this series (48%) compared to studies conducted at urban tertiary care centers (16.1%, 25%) [11, 12]. A previous study performed at our urban, tertiary eye care center also reported a lower proportion of DFK (23.5%) [5]. This study describes the clinical course and outcomes of 108 eyes of DFK from a peripheral rural center in northern India.

The study center was situated in an area predominantly composed of sugarcane and paddy fields, which likely contributed to a higher incidence of trauma associated with vegetative matter in this cohort (71.3%), compared to the findings reported by Ganesan et al.(33.3%) and Kumar et al. (56.1%) [7, 11].

Our study reported a higher healing rate (93.5%) compared to previously published literature, where Chaudhary et al. and Ganesan et al. observed healing rates of 82.1% and 79% respectively [6, 7]. Early presentation in treatment-naïve cases, along with debridement or superficial keratectomy of the plaque may have contributed to this favorable outcome.

The use of prior topical steroids was an indicator of poor outcomes, as significantly more of these patients required a major surgical intervention (21.4%). However, amongst the other 78.6% of patients with a history of steroid usage who healed medically, there was no difference in the time taken to heal. This is consistent with published literature [1, 7, 11].

Notably, 27% of the patients with a treatment history were using antifungal medication. However, they sought medical care at our center because of a poor response to treatment. It can be hypothesized that the poor therapeutic response may be due to inadequate dosing and insufficient penetration of the drug through the thick infective plaque. Theoretically, therapeutic removal of the plaque should hasten the healing response [1]. In this series, 93.5% of patients underwent debridement or superficial keratectomy at every visit as it is a part of the standard operating procedure of the institution, and had complete healing. The remaining 6.5% of patients were not amenable to debridement, given a perforation or a corneal melt. The latter group of patients underwent a major surgical procedure.

Ganesan et al. also reported that a therapeutic debridement in 17% of patients with a pigmented plaque led to complete healing in these patients [7]. Beyond the scope of this study, it would be interesting to compare the duration of healing amongst patients treated with frequent therapeutic debridement versus patients treated without a therapeutic debridement.

Irrespective of the size, a majority of the ulcers were superficial and healed medically. The longer time to heal for larger ulcers was attributed to a higher chance of the formation of a neurotrophic-like defect. On the other hand, deeper ulcers had a significantly higher chance of requiring a major surgical intervention. This finding of formation of neurotrophic-like defect may represent a sequela of the intensive treatment regime for fungal keratitis or result from the release of pro-inflammatory markers, extracellular enzymes, and/or mycotoxins from the dying fungi [13–15]. During this stage, it is important to reduce the frequency of antifungal drops while increasing frequency of lubricant drops. Further research is required to determine the role of such mycotoxins or pro-inflammatory markers on the clinical course of fungal keratitis.

Clinically, poor outcomes were associated with corneal melt or perforation at presentation (OR = 42), vascularization (OR = 14), and the presence of a hypopyon (OR = 2.33) among the clinical characteristics. Previous treatment with antibiotics or steroids, as well as larger ulcer size and greater ulcer depth, were also identified as risk factors for poor outcomes. Several cases in these categories eventually required TPK/evisceration, whereas none from the reference group did (OR = infinity). These findings are consistent with those of Garg et al. [1].

The strength of this study lies in the source of its data from a rural secondary care center catering to the more susceptible population and the high incidence of DF in our cohort as compared to previously published studies. Furthermore, our data includes a greater proportion of treatment naïve ulcers unlike many studies based on tertiary centers that predominantly receive referred or recalcitrant cases. In this regard, our study represents real-world data from the underrepresented rural population, and the outcomes more accurately describe the natural course and response of treatment in DFK. We were not able to identify the subspecies of DF due to resource constraints. Whether it has any bearing on clinical course and outcomes can be an area of further research.

To conclude, DFK cases demonstrate a high healing rate of 93.5% with medical therapy and minor procedures. Although healing can be prolonged, appropriate treatment combined with complete plaque removal often results in favorable outcomes. Poor prognostic factors include the presence of large and deep infiltrates, hypopyon, and a history of prior corticosteroid use.

## Data Availability

Available with the corresponding author.
